# Performance management in primary healthcare: Nurses’ experiences

**DOI:** 10.4102/curationis.v43i1.2017

**Published:** 2020-04-30

**Authors:** Cynthia Z. Madlabana, Inge Petersen

**Affiliations:** 1 School of Applied Human Sciences, Discipline of Psychology, University of KwaZulu-Natal, Durban, South Africa

**Keywords:** performance management, performance appraisals, human resources for health professional nurse, primary health care, national health insurance, integrated clinical services management, person-centred care

## Abstract

**Background:**

The use of the performance management (PM) system is highly contested by public servants in South Africa, although its value as essential to the appraisal and management of staff is undeniable.

**Objectives:**

The aim of this study was to explore nurses’ perceptions and experiences of the PM system at primary healthcare (PHC) facilities in relation to the current health system’s reforms.

**Method:**

An exploratory, descriptive and qualitative design was utilised. Participants were selected through purposive sampling. A semi-structured interview tool was used to collect data from 18 nurses in four sub-districts of Dr. Kenneth Kaunda district in the North West province. Data were analysed through thematic analysis.

**Results:**

The findings of this study confirmed that PM is implemented to some extent. However, various loopholes in its implementation threaten the accuracy and transparency of the system and leave it vulnerable to perceived organisational injustice and unfairness, with the objectivity of the system questioned. The limitations of the current PM system revealed by this study include (1) the lack of alignment with current health system reforms towards comprehensive and integrated care that demands person-centred care; (2) the system’s usefulness for career progression, performance improvement and rewarding exceptional performance.

**Conclusion:**

Performance management is inadequately applied in PHC facilities at district level and needs to be realigned to include the appraisal of key attributes required for the current health system’s reforms towards comprehensive and integrated care, including the provision of person-centred care, which is central for responding adequately to South Africa’s changing disease profile towards multi-morbidity.

## Introduction

Currently, health systems reforms are underway in South Africa, notably the introduction of National Health Insurance (NHI) and the re-engineering of primary healthcare (PHC) to promote integrated clinical services management (ICSM) of acute and multi-morbid conditions. Given the increase in the prevalence of multi-morbid conditions (Mayosi & Benator 2014), the vertical disease-oriented approach to care is no longer suitable and there is a need for a shift in orientation towards collaborative team-based person-centred care (PCC) to understand the patient holistically as well as engage them in their own healthcare (Maseko & Harris 2018). Person-centred care is defined as an approach to healthcare that emphasises communication with patients, being cognisant of the issues that are beyond any single disease/condition (Jardien-Baboo et al. 2016). Accordingly, organisational systems and processes such as performance management (PM), organisational culture and organisational strategic objectives should be harmonised to align with these reforms. Poor human resource management (HRM) methods and practices in the healthcare system have been found to threaten the successful implementation of quality healthcare in South Africa (Republic of South Africa 2012a). Furthermore, these processes must be managed appropriately to encourage a shared vision, inspire health workers and build a culture of performance that drives the entire health system towards a common purpose.

Given that nurses are at the frontline of healthcare delivery, constituting 80% of health workers of public healthcare providers nationally (Rispel, Moorman & Munyewende 2014; Statistics South Africa 2017) , the need to ensure that nurse-related HRM practices are aligned with the current reforms is not only important but indeed essential.

According to Rispel (2015), South Africa faces a nursing crisis that is characterised by personnel shortage, a declining interest in the profession, staff disengagement and lack of resources. In addition, the nursing profession has come under attack for poor service delivery (Republic of South Africa 2012b). In an attempt to attract and retain nurses within the South African healthcare system, as well as to improve quality of service provision, recently increased attention has been paid on how HRM processes and outcomes influence nurses’ experiences, attitudes and behaviour at workplace and ultimately the quality of care they provide (Mayosi & Benatar 2014; Rispel 2015; Rispel & Barron 2012).

Performance management system is an essential component of HRM. It is particularly important within healthcare organisations as it is a formal process that determines progress on expected outcomes versus actual outcomes (Moradi et al. 2017). Health human resource (HR) practitioners and nurse managers are confronted with the need to develop HR practices that support behavioural changes and promote support for structural changes such as the NHI and ICSM that are associated to person-centred care. Previous studies have identified the following factors as essential in the development of a PM system to promote job satisfaction and improve the delivery of care. Firstly, individual factors that reward performance and manage underperformance in a manner that is clear, accurate and fair (Lee & Steers 2017; Steers & Lee 1982). Secondly, management styles that promote supportive leadership and champion good people management practices (Boaden et al. 2008). Thirdly, promotion of teamwork and conflict reduction strategies (Skinner et al. 2018). Fourthly, organisational systems that are aligned with the promotion of person-centred care and empowering organisational environment which allow for decision latitude and job control (Albrecht et al. 2015; Van De Voorde & Beijer 2015).

However, performance management and development system (PMDS) generally is highly contested and perceived as lacking transparency and accountability (Republic of South Africa 2007b) and vulnerable to bias and unfairness (Ghauri & Neck 2014). The South African public service PM system has been criticised for being ‘generally poorly implemented’, with further research necessary to understand how best to promote good practices and competency as well as the role of continuous professional development for strengthening human resources towards the provision of quality healthcare (Kabene et al. 2006; Mello 2015).

In light of the paucity of evidence on nurses’ perceptions and experiences of the PM system in PHC, understanding nurses’ perceptions and experiences of the PM system is a vital first step to understand how this system could be improved to promote quality of care in the context of the health system’s reforms at PHC level.

### Aims and objectives

This study was aimed to explore nurses’ perceptions and experiences of the current PM system in relation to the changes in their roles and functions as a consequence of the current health system’s reforms in South Africa. More specifically, the objectives of the study were to explore nurses’ perceptions and experiences of the PM system and its influence on quality of care within the context of re-engineered PHC, NHI and ICSM that demand PCC. In doing so, the authors describe actions that PHC facilities could consider towards improving the use of the PM system to cultivate a culture that fosters quality of care within the context of re-engineered PHC, NHI and ICSM that demand PCC.

### Definition of key concepts

#### Integrated chronic services management

Integrated clinical services management is a system of managing care that provides for an integrated method for prevention, treatment and care of chronic patients at primary healthcare level. It aims to ensure a transition towards ‘assisted’ self-management within the community. This could be achieved through the adoption of a patient-centric approach to healthcare that encompasses the full value chain of continuum of care and support (Bodenheimer & Bauer 2016).

#### National Health Insurance

National Health Insurance is a health financing system designed to pool funds to provide access to quality affordable *health* services for all South Africans based on their *health* needs, irrespective of their socio-economic status. Matsoso and Fryatt (2012) have reported that the purpose of NHI is to achieve universal health coverage (UHC) and establish a unified health system.

#### Quality of care

Quality of care is defined as a process of improving services in health systems. This is achieved by applying safe, effective, person-centred, efficient and equitable services to achieve desired health outcomes (World Health Organization 2006).

#### Performance appraisal

A performance appraisal system refers to a period of the evaluation of employee’s performance against set expectations. It is described by Aguinis (2009) as a systematic description of an employee’s strengths and weaknesses.

#### Performance management system

According to DeNisi and Murphy (2017):

Performance management refers to the wide variety of activities, policies, procedures, and interventions designed to help employees to improve their performance. These programs begin with performance appraisals but also include feedback, goal setting, and training, as well as reward systems. (p. 1)

#### Performance management and development system

The PM system used by the South African public services is referred to as a PM and development system. It was implemented in 2012.

#### Re-engineered primary healthcare

According to Schaay et al. (2011), the chief change in the healthcare system is the re-engineered PHC reform initiative. Re-engineered PHC aims to strengthen the district health system with greater emphasis on quality of service delivery, highlighting the social determinants of health.

## Research methods and design

### Research design

An exploratory qualitative design was used to explore professional nurses’ (PNs) perceptions and experiences of the current PMDS. Moreover, it is applied to describe nurses’ perceptions and experiences on how the PM system can be used to cultivate a culture that fosters quality of care.

### Research site

This study was conducted in the Dr Kenneth Kaunda district of the North West Province of South Africa. The municipality has four sub-districts, namely, City of Matlosana, Maquassi Hills, Ventersdorp and Potchefstroom (renamed Tlokwe). The Dr Kenneth Kaunda district has an estimated total population of 807 252. Health services are delivered by one regional hospital, two district hospitals, nine community health centres, 27 clinics (North West Department of Health [NWDoH] 2018). Economically, North West’s strongest economic contributions are in mining and agriculture sectors.

This district was chosen because it was a pilot district for NHI and ICSM. Given that the health system’s reforms necessary for the introduction of NHI and ICSM were being piloted in this district, it provided an opportune study site to meet the objectives of the study.

### Selection of participants and data collection

Upon receiving appropriate ethical clearance, operational managers of all facilities in each sub-district were contacted to schedule a suitable time for the visit of research team. Nurses were informed about the purpose of the research study and invited to participate. Professional nurses were recruited employing a purposive sampling approach. The sample constituted of professional nurses registered with the South African Nursing Council (SANC). Professional nurses are described by the *Nursing Act* (Act no. 33 of 2005) Section 30 (1) (Republic of South Africa 2005). It defines a professional nurse as follows:

[*A*] person who is qualified and competent to independently practice comprehensive nursing in the manner and to the level prescribed and who is capable of assuming responsibility and accountability for such practice. (p. 34)

More nurses who were recruited worked in City of Matlosana sub-district, given that it is the largest sub-district followed by Potchefstroom. The distribution of participants is shown in Table 1.

**TABLE 1:** Participation distribution per sub-district.

Name of sub-district	Number of professional nurses interviewed	Percentage (%)
Matlosana (M)	8	44
Potchefstroom (Tlokwe) (P)	4	22
Ventersdorp (V)	3	17
Maquassi Hills (MH)	3	17
Total number of participants	18	100

Eighteen interviews were conducted with professional nurses using a semi-structured interview schedule. The interview questions were formulated using the guidelines provided by the Public Services’ Employee Performance Management and Development System framework (Republic of South Africa 2007a). The interview questions therefore included an understanding of performance standards, performance measures, feedback, leadership and improving quality of care. In addition, the interview schedule also included pertinent questions on working in teams and promotion of change management. The interviews were conducted in English; however, occasionally participants communicated in Setswana and IsiZulu; for such cases, an independent translator was appointed to translate the interviews into English. The interviewers included the first author and a research assistant studying for Masters in Organisational & Industrial Psychology. Both possessed the necessary comprehension of each language. Participants were predominately Setswana-speaking. All interviews were audio-taped, translated (where necessary) into English and thereafter transcribed.

### Data analysis

Thematic analysis was used by the first author to analyse the data obtained from semi-structured interviews. This approach is prominent in qualitative research for its accessibility and theoretical flexibility as it identifies and interprets patterns of meaning across data content (Braun & Clark 2006, 2014). Moreover, this analytical framework provides rich and detailed information from data. The process of analysis involved systematically reading the transcripts. In doing so, common themes were identified from the actual words of participants (Braun, Clark & Terry 2014). Furthermore, the process of refining themes and determining coherent patterns was adhered to in order to ensure that the researcher accurately captured the nurses’ perceptions and experiences as communicated by the data set. The process of analysis concluded by identifying attributes and conclusions drawn from the themes based on supportive literature and related previous research (Smith 2015). This process required a concise, logical and non-repetitive account of the data, and appropriate literature was used to confirm and/or challenge the research findings.

### Trustworthiness

Measures to ensure trustworthiness as highlighted by Lincoln and Guba (1985) formed the present study. These measures are, namely, credibility, transferability, dependability and confirmability. The concept of trustworthiness refers to attaining knowledge and understanding of the true essence and characteristics of the phenomenon and thus providing reliable accounts of data (Marshall & Rossman 1995). To achieve credibility, the researcher took steps to validate the findings through more than one coder and comparing coded data and using thick descriptive data to support resultant themes. Transferability was assured by using a sampling strategy to ensure a good spread of participants from each sub-district of Dr Kenneth Kaunda district. It was further ensured by describing the research processes in detail to enable replication of the study in another setting. Dependability was achieved by external control. The study was part of a larger research project, the supervisor of the project had oversight authority to oversee the study and ensure its dependability. All interview materials (such as audiotapes, transcripts, etc.) are stored safely in a locked cupboard and only available to the research team involved in the analysis of the data. An audit trail of the data collection and analysis was carefully documented for the purpose of audit trail (Lincoln & Guba 1985). Confirmability was ensured by both researcher and research supervisor checking and rechecking the emergent themes obtained from the data; the use of thick descriptive data to support emergent themes; and comparing data with previous research findings (Nowell et al. 2017).

### Ethical considerations

Ethical clearance was obtained from the Biomedical Research Ethics Committee (BREC) Board at the University of KwaZulu-Natal (reference number: BE084/16). Once the provisional approval was granted, gatekeeper approval was obtained from the North West Department of Health (NWDoH): policy, planning, research, monitoring and evaluation upon obtaining approval from NWDoH, full ethical clearance was granted by BREC. Ethical protocols, including anonymity and confidentiality, were adhered to. To safeguard participant information, all personal identifying information was removed from the data. Participants were informed that participation in the study was voluntary and they have the right to withdraw from it at any point without any consequences. All participants provided written informed consent to participate in the study. Only the research team had access to collected data. All audio-taped recordings and copies of interview transcripts are stored in a secure vault for a maximum period of 5 years as per ethical stipulation. Thereafter, they would be destroyed and the recordings would be erased and deleted. Participants could request a copy of the final research report.

## Results

### Sample profile

The majority of professional nurses were black (89%; *n* = 16) and females (83%; *n* = 15), aged 41–50 years (55%; *n* = 10). Furthermore, most professional nurses indicated possessing a diploma in nursing (72%; *n* = 13). The professional nurses’ years of experience ranged from less than 5 years’ work experience (50%, *n* = 9), and between 6 and 10 years (44%; *n* = 8).

The themes that emerged from the data as shown in Table 2.

**TABLE 2:** Themes and subthemes.

Themes	Subthemes
1. Importance of PMDS in healthcare	PMDS as a strategic, administrative and developmental managerial tool.
2. The system is flawed	Experiences of poor implementation of PMDS Inconsistencies in the application of PMDS across sub-districts. Weak Accountability
3. Rewarding Performance	Rewarding system Favouritism
4. Key considerations for effective performance management	Re-evaluating administration of the system Providing adequate training on PMDS for complete staff Facility managers’ competency and capacity to evaluate nurses’ performance Team dynamics and their consequences
5. Experience of health system’s reforms	Change is good and bad
6. Improving quality of care	Quality of care requires staff, resources and time

PMDS, performance management and development system.

The main themes and subthemes reflect on the nurses’ perceptions of the current PMDS within PHC healthcare settings. Although quite a good number of nurses mentioned PMDS as a vital HR component, many argued that it could become more relevant if it accurately measures performance and is used for development purposes. McDermott and Keating (2011) and Hyde et al. (2013) suggest that, currently in practice, the PMDS is not used effectively.

Each theme and subtheme is discussed comprehensively below.

Data were coded according to occupation and interview number. As indicated below: PN1–PN18, where PN refers to professional nurse and 1–18 refers to interview number.

#### Theme 1: Importance of performance management and development system in healthcare

This theme covered the importance of PMDS; it includes the purpose of PMDS and its current use in PHC health facilities.

**Performance management and development system as a strategic, administrative and developmental managerial tool:** The majority of participants perceived one of the goals of PMDS as being to evaluate performance for the purpose of identifying areas of development in nursing practice and thus assisting nurses in improving the quality of care rendered to patients for the purpose of meeting strategic goals towards better health services delivery. This is captured by the below-mentioned participant who mentioned the development focus of PMDS:

‘It is developing because where you are able to see gaps, they are able to come back to you and say, you lack knowledge on this and that and then take you for training.’ (PN14, female, 4 years of experience)

It was also observed that if applied correctly, the PMDS has the potential to help improve quality of service delivery through monitoring and evaluating performance for achieving national strategic health objectives. These aspects were mentioned by the participants mentioned below:

‘It is like auditing the staff, like how far did you go, are you in line with the working environment and the guidelines etc.’ (PN16, female, 11 years of experience)

‘It is a good thing to also control performance and to reward people that need … rewarding.’ (PN11, male, 6 years of experience)

The above-mentioned participants demonstrated that the PMDS has a quality assurance aspect whilst also providing incentives for good performance. The remunerative incentive emerged as a key positive factor for the usefulness of PMDS for a number of participants:

‘For remuneration, for payment purposes and rewards, that is the only important part about it. Somehow it encourages us, “If you work hard you will be rewarded”. You become more interested in doing your job and wanting to do more.’ (PN14, female, 4 years of experience)

The use of PMDS to incentivise performance is discussed in more detail under theme 3.

#### Theme 2: The system is flawed

This theme covers nurses’ perceptions and experiences concerning how the PMDS is implemented at district level. It highlights the inconsistencies in its application across sub-districts as well as its weak accountability control measures.

**Experiences of poor implementation of performance management and development system:** The majority of participants complained about the poor implementation of PMDS. For example, participants articulated that whilst the appraisal process was meant to acknowledge and reward diligence as reflected in the previous theme, this was not always the case:

‘It is failing us because even though we work … you end up being at the same salary level for so many years.’ (PN1, female, 8 years of experience)

The participant below also mentioned that as a result staff were reluctant to participate in the process. Furthermore, this participant reported there were no serious repercussions if staff chose not to complete the appraisal at all, thus demonstrating how the application of PMDS was compromised:

‘Everybody is just reluctant …. I don’t see any progress in this system … even if we don’t write it … nothing is done….’ (PN5, female, 7 years of experience)

Interestingly, another participant (professional nurse 16) also passionately advocated against the use of PM, citing its idealistic aims that create animosity, given the unrealistic demands associated with it and with it having the potential to create an inconducive working environment that is contradictory to the vision of the system:

‘The PMDS is out of order, it is unrealistic … it is not working, the targets, its numbers, it is just imaginative numbers … The PMDS is creating some form of hatred to some people who are working and are not getting it …’ (PN16, female, 11 years of experience)

Another related issue was the unrealistic targets set by NWDoH which result in nurses feeling undermined when they do not reach their targets:

‘This year you must treat a hundred patients, critical patients who are involved in an accident. Then you find you only have ten patients who are involved in an accident. How are you supposed to do that? Are you now supposed to spread the message to those people to please get into accidents? That is the problem with PMDS. They are not applicable and realistic. The problem with the PMDS is the targets.’ (PN16, female, 11 years of experience)

**Inconsistencies in the application of performance management and development system across sub-districts:** Another complaint to emerge related to inconsistencies in the scoring and rewarding of the PMDS across sub-districts, with more than half of the participants mentioning this was an issue:

‘They are not measured in the same way … With remuneration … same – if I compare myself with a professional nurse with the same experience as mine maybe Klerksdorp, we are doing the same thing like on a daily basis. She will be recognized and I won’t be …’ (PN14, female, 4 years of experience)

**Weak accountability:** Most nurses mentioned a lack of process to hold those not providing accurate critique to account for their performance. Half of the participants mentioned that weaknesses in the implementation of PMDS compromised the legitimacy of the system. For example, nurses claimed they were allowed to refuse to participate without repercussions; others were writing anything to avoid being penalised and recycling submissions:

‘Last year I refused to write …’ (PN3, female, 3 years of experience)

‘You just write to get finished, just to get it done because when you don’t write they will always be on your neck, putting pressure on you saying, “We need your PMDS we’re going to penalize you if you don’t write”.’ (PN4, female, 1 year of experience)

‘We are only told to write it, then we copy from others. Then we ask others how you write it, so we only get information from other staff.’ (PN3, female, 3 years of experience)

#### Theme 3: Rewarding performance

The majority of participants repeatedly expressed perceptions and experiences of great dissatisfaction with the rewarding system and further highlighted that the validity of PMDS was compromised by favouritism at facility level.

**Rewarding system:** Whilst participants acknowledged the potential of PMDS as a system for motivating them to perform better, the majority of them mentioned that there was a strong sense across the board that the manner of distributing rewards was not justified:

‘I want them to treat us equally … we are all going the extra miles … they should also give us some bonus for our hard work … we are working very hard compared to other clinics.’ (PN3, female, 3 years of experience)

The below-mentioned participant argued that in instances where staff members did not receive a bonus, feedback must be provided on why they did not qualify:

‘They [*facility managers*] should explain why you didn’t qualify for a performance bonus … [*and others do*]. We’re coming to work every day doing what you are supposed to do.’ (PN15, female, 7 years of experience)

The majority of participants identified the need for the PMDS to measure performance accurately, consistently and without any ambiguity. In doing so, these participants expressed great dissatisfaction with how performance decisions were made. Following comments attested to this:

‘The people that get it [*performance rewards*] are the ones who are not working …’ (PN3, female, 3 years of experience)

‘It is not benefiting the people on the ground who are actually doing the job.’ (PN16, female, 11 years of experience)

Like the above-mentioned participants, the following two participants also confirmed the lack of uniformity, and discrepancies, and questioned the fairness of the system:

‘Sometimes those that are absent and dodging at the end of the year they are getting [*performance rewards*] …’ (PN9, female, 2 years of experience)

‘People that are writing PMDS are getting all these good remarks but they are not doing anything in the clinic.’ (PN11, male, 6 years of experience)

Apart from the monetary rewards described above, some participants also mentioned the need for non-monetary plans of recognition and appreciation for hard work that could boost self-esteem and commitment:

‘To be appreciated in a way, I don’t say they should give money or whatever just to say, “Hey you have done well today”. People’s self-esteem is being built up. At least I am being appreciated; it doesn’t mean it is all about money.’ (PN7, male, 5 years of experience)

**Favouritism:** Almost all the participants suggested that the PMDS was riddled with favouritism at facility level. Participants noted the following as they interacted within their own work environment:

‘The manager does play a role … they do also have their favourites, it is not fair.’ (PN11, male, 6 years of experience)

‘It is favouring other people … it is the same people that always get PMDS [*rewards*].’ (PN15, female, 7 years of experience)

Other participants indicated that favouritism had a negative impact on performance and how it created conflict between employees:

‘It causes friction. It breaks the spirits because if someone gets a reward and I don’t get it when we are in the team. What’s the difference?’ (PN9, female, 2 years of experience)

‘I don’t think PMDS is working … people will tell you that if your manager favours you, it will benefit but if your manager doesn’t favour you then you don’t benefit.’ (PN17, female, 5 years of experience)

Professional nurse 16 cited managers for being responsible for initiating PM initiatives as well as having maximum benefits through favouritism.

‘It has some sort of favouritism … the managers are getting the PMDS [*rewards*] but the ones who are working, who are hands on are not getting the PMDS [*rewards*].’ (PN16, female, 11 years of experience)

Overall, participants felt that favouritism ultimately had devastatingly negative consequences for positive work outcomes such as motivation, job performance and team collaboration.

#### Theme 4: Key considerations for effective performance management

This theme highlights nurses’ opinions on key considerations for effective PM. These considerations include the following: (1) re-evaluating the administration of the system; (2) providing adequate training on PMDS for complete staff; (3) evaluating facility managers’ competency and capacity to evaluate nurses’ performance and (4) team dynamics and their consequences.

**Re-evaluating administration of the system:** Many participants suggested the need for a change in the current PMDS, wanting it to be aligned with PMDS within the district hospital settings. This was because the latter PMDS was perceived to be more user-friendly, with nurses merely having to rate themselves on certain criteria using tick boxes as opposed to writing in the format used by the PMDS in PHC facilities:

‘I came here from the hospital, and usually the hospital, they had already, written it [the appraisals]. It was just for you to maybe tick [rate] yourself and then immediately when I came here I heard that everyone has to write the PMDS. No one showed me how to do it.’ (PN5, female, 7 years of experience)

‘If you can check Potch hospital, they are not writing they are scoring themselves. They get everything written with a column on the right to score. So you tick.’ (PN14, female, 4 years of experience)

**Providing adequate training on performance management and development system for complete staff:** The majority of participants advocated for compulsory training on PMDS for the complete staff involved. They expressed that they did not feel confident about their capabilities to use the system, and that is why they felt vulnerable to making errors. It was suggested that the training of staff should focus on the process that raters and ratées should follow during the PM cycle, including establishing performance objectives, performance appraisal mechanisms and communication of performance appraisal feedback. These sentiments were shared by the majority of participants and exclusively expressed by the participant below:

‘We don’t even know how we should write PMDS because always when we write the PMDS, they will always tell you that this is the wrong way; this is not the correct way. But they have never conducted a workshop or training, so that we can all be on board as to what they expect from us.’ (PN4, female, 1 year of experience)

Participants also indicated that poor communication of performance appraisal feedback impacted negatively their performance:

‘Sometimes they don’t even tell you; you will be scoring yourself, take the PMDS, to your supervisor and sometimes she will be scoring you and then attach the signature then send the forms to HR, without knowing the percentage you got.’ (PN4, female, 1 year of experience)

Poor feedback of the performance appraisal process was reflected in the participant’s following statement:

‘If it was useful [*the feedback*], you would know what to write in the next PMDS and be sure that this is the correct way of writing it. They should call us individually and sit you down, discuss everything that you wrote so you know what it is that you did good [*well*] and what is it that you didn’t do well. Because after presentations, no one is coming back with the feedback.’ (PN14, female, 4 years of experience)

**Facility managers’ competency and capacity to evaluate nurses’ performance:** Many participants questioned whether facility managers had the time and competency to carry out PMDS effectively. They suggested that nurse managers were involved in much of administrative tasks that consumed substantial amount of their time, compromising contact time with nurses. The following statement illustrates the participant’s views:

‘The managers. They don’t have time. Even when you have a problem or you sitting down with her, she will just tell you this and this and then you must go … So we are not satisfied about this PMDS, the evaluation, the improvement and management.’ (PN2, female, 4 years of experience)

Some participants suggested that nurse managers did not adequately represent the performance of professional nurses during panel evaluation committee meetings when individual and facility performance rating are evaluated:

‘I don’t think she is doing enough when she presents us at the panel … Maybe she is not doing enough to prove that, “No, this person is really a hard worker”.’ (PN15, female, 7 years of experience)

Other participants stated that managers were not supportive in assisting them to improve performance and were only concerned with meeting targets. Thus, managers were often not proactive in addressing under-performance:

‘They are not supportive, they are concerned about the numbers. They address the problem as it comes. It is only when something happens afterwards, they will come in and say “Why did this happen?” I have seen that at the top, that if the sub-district is not doing well, they come down and they put the pressure on us.’ (PN10, female, 1 year of experience)

**Team dynamics and their consequences:** The majority of participants also perceived PMDS as largely individualistic in nature and consequently not encouraging teamwork, which was regarded as essential to achieve facility performance and improve the quality of collaborative teamwork, which underpins the chronic care model. The existing PMDS system was viewed as working against collaborative teamwork and the need for the PMDS to be aligned with organisational changes promoting teamwork was thus highlighted.

‘It [*is*] dividing the staff. Currently, we are trying to work as a team, but if we are working as a team and then I alone get the PMDS or something but we are doing the same thing together I think that is separating the team work so someone will start only concentrating on the things she is supposed to achieve to get the performance bonus.’ (PN15, female, 5 years of experience)

#### Theme 5: Experience of health system’s reforms

This theme provides nurses’ experiences with change and how the change is managed. The current changes were narrated in relation to the implementation of re-engineered PHC, NHI and the promotion of quality improvement.

**Change is good and bad:** Some nurses perceived current changes in a positive light, whilst others protested that there were too many changes all at once as indicated in the following statement:

‘Too many changes at the same time ….’ (PN9, female, 2 years of experience)

In addition, a number of participants indicated that the changes had resulted in increased workload for limited staff as suggested in the following statement:

‘I feel like those changes are piling up the workload on us … they actually put more pressure on us.’ (PN17, female, 5 years of experience)

‘It adds more work load … these changes of policy and these guidelines. You will be knowing this policy and mastering it and then 2 months later they introduce another one … the other one is more work so it is influencing the queuing, the waiting times and the quality care of the patient because now you have to do more on the patient and the writings.’ (PN14, female, 4 years of experience)

The training provided to implement the changes was also deemed insufficient to provide them with the necessary competencies to render quality care as explained below:

‘Trainings are not enough, they do trainings for maybe 3 days and then they expect us to do this thing thoroughly and perfectly so …’ (PN14, female, 4 years of experience)

On the contrary, these negative views regarding the changes were countered by some of the nurses sharing positive narratives on change, particularly in relation to the introduction of guidelines and manuals as suggested below:

‘They really help … you get a patient, you don’t know how you really going to treat this patient, he comes in having these symptoms, you can just go to your guidelines and then you will be checking the different symptoms your patient has.’ (PN13, male, 5 years of experience)

‘It has improved the way I have been doing my job because now we are now having guidelines instead of me wasting time calling the doctor or someone to give me advice, I just open up the book and see that the patient I can help her this way so it saves a lot of time.’ (PN8, male, 3 years of experience)

Notwithstanding these positive aspects of the changes, the majority of participants found that these changes were not managed effectively, with the barriers being mostly organisational – such as lack of resources and staff. Such organisational factors are discussed in length in the next theme.

#### Theme 6: Improving quality of care

This theme detailed nurses’ perceptions on how to use PM system to cultivate a culture of improving the quality of healthcare delivery and factors that need to be considered in this regard.

**Quality of care requires staff, resources and time:** The majority of participants complained of staff shortage, with pressure to see many patients, and that this compromised the quality of care they were supposed to provide:

‘With quality … we try our best but the numbers increase and what happens is that we open 08:15 up until 16:30. The clinic can be full up until 16:00 so we hurry up because we want to go home in time. You won’t render quality services to your patients you just want them out of here.’ (PN13, male, 5 years of experience)

‘Work overload … so many patients to see, you end up not seeing one patient in totality. We run to push the line, making the hall empty, helping people sitting outside so that we can go. So that affects the quality of care we are rendering the patients.’ (PN14, female, 4 years of experience)

One nurse, however, had a different view, highlighting that quality should not be compromised, regardless of long queues. This participant noted the need for nurses to pay attention to individual cases, highlighting the importance of nurses providing health-promoting messages, although she was not optimistic about patients’ willingness to receive such care:

‘We cannot just hurry up because the queue is long. If you see that this patient needs attention, you just do what we have to do. We have to give quality … We encourage patients to keep healthy and also the information we are giving our clients, it must, at least, help them to change their lifestyle … In our clinic, patients are discouraged to come because of long queuing. Sometimes, even if you can give them information, they are not listening to go. I think our community lost hope in [*the*] health system.’ (PN9, female, 2 years of experience)

Some participants indicated the need for greater support for the challenges faced by nurses, indicating the need for greater awareness on the part of management in this regard:

‘I think support and communication would be the best, if management can come down [*sic*] and maybe look at the work that we do, so to understand how many patients we administer.’ (PN16, female, 11 years of experience)

Many participants further indicated that the current PMDS overemphasised meeting targets (being outcome-based) and the neglect of consideration on the quality of care provided (behavioural-based), which is central to PCC. This is further indicated by the following participant:

‘The PMDS is more about the numbers, not the quality of work you are doing. It has nothing to do [*with*] the people, but the numbers!’ (PN16, female, 11 years of experience)

## Discussion

### The importance of performance management and development system in healthcare settings

The vast majority of participants identified the need for a PM system in healthcare settings. They understood that its main purpose was to meet up with and evaluative developmental objectives, and that the value of PMDS dwells in its potential to provide feedback that could be helpful in improving their job performance and the provision of quality care. This developmental ethos in managing performance is supported by Lutwama, Roos and Dolamo (2013), who identified it as one of the three main functions for a PM system (the others being strategic and administrative).

### Monetary and non-monetary rewards

Both monetary and non-monetary rewards emerged as important aspects of performance appraisal in this study. The majority of participants expressed that the profession was extremely stressful, and lack of recognition and rewards was one of the major reasons for their job dissatisfaction. Although monetary rewards were reported to be important to improve job satisfaction and retention, the importance of other forms of recognition and acknowledgement also emerged as important. Other forms of appreciation, such as recognition for daily progress, were reported to enhance positive attributes such as dedication, hard work and self-esteem. Abualrub and Al-Zaru (2008), who conducted a study on job stress, recognition, job performance and intention to stay at work amongst Jordanian hospital nurses, found a direct and buffering effect of recognition of nurses’ performance on job stress and the level of intention to stay at work. Locally, the importance of recognition for outstanding performance as well as other achievements is also supported by Mokoka, Oosthuizen and Ehlers (2010), who found that both monetary and non-monetary rewards were important for improving retention of professional nurses in South Africa from a nurse manager’s perspective.

### Evidence of a system that is poorly implemented

The nurses highlighted that the PMDS was poorly implemented. Nurses complained that the way in which PMDS was implemented failed to truly capture performance, did not provide feedback on remedial steps to improve poor performance and did not promote accountability or set realistic performance targets. Mone and London (2018) suggest that if true performance is not captured accurately or consistently, it would decrease the natural motivational climate to enhance performance. On the contrary, if the system is implemented correctly, it would facilitate identification of non-performance and implementation of remedial interventions to improve performance.

Furthermore, the participants in this study perceived the system to be implemented unfairly and lacking impartiality – with the respondents questioning whether those receiving rewards truly deserved them. Monetary gain as an incentive was reported to fuel distrust and promote favouritism, with only few earning monetary rewards. Such beliefs are in line with previous literature that has investigated the perception of PM in the public sector of South Africa (Makamu & Mello 2014; Mello 2015; Swaartbooi 2016). Daskin (2013) found similar experiences in the hospitality industry, with favouritism having the potential to create distrust and causing diligent performers to disengage from the process. Favouritism has been found to be disruptive for productivity and staff morale, creating conflict between employees, and affecting negatively on motivation, job satisfaction, job performance and team collaboration (Alotaibi, Paliadelis & Valenzula 2016; Isaed 2016; Platis, Reklitis & Zimeras 2015). The need for nurse managers to be trained in the negative implications of favouritism in the PMDS process is thus highlighted.

Owing to the perceived unfairness of the system, not feeling competent in how to complete their side of PMDS, as well as lack of feedback on their performance and how it could be improved, nurses purportedly did not take the system seriously or understand why it is necessary (Du-Plessis 2015). They commented that they participated in the process only to avoid being disciplined if they did not comply. Although the tools and processes of PM are based on sound principles, how they are implemented and utilised is contentious (Mboweni & Makhando 2017). One of the greatest challenges in literature on PM systems and performance appraisal involves employees contesting its usefulness in fostering self-development and promotion (Adler et al. 2016; Mone & London 2018). The need for management training for the purpose and use of PMDS to ensure that it is implemented as intended is again highlighted.

### Quantity over quality

Nurses in this study also mentioned that the overemphasis on outcome-based measures of performance compromised attention to quality of care and PCC. There were no incentives for professional nurses to practise PCC. Behaviour-based measures of performance that could be used to promote this approach to care were neglected. Examples of such evaluation include measuring the relationship between patient and nurse, patients and nurses agreeing on patient problems, and efforts towards evaluation of medical and other interventions to resolve or improve patient care. Instead, the PMDS encouraged nurses to spend less time on each patient to achieve their targets and to ensure that all patients visiting healthcare facility are served (Hanefeld, Powell-Jackson & Balabanova 2017; Petersen et al. 2006).

In the context of current reforms underway in PHC, the PMDS presents as a valuable tool that could assist in ensuring implementation of these reforms. This is especially the case in relation to reorienting staff to providing a person-centred team-based collaborative care necessary for treating multi-morbid chronic conditions that commonly present at PHC because of the clashing human immunodeficiency virus (HIV) and non-communicable diseases (NCD) epidemics (Kengne & Mayosi 2014). Awases, Bezuidenhout and Roos (2013) warned that the performance of health workers is linked with productivity, whilst provision of quality care within healthcare facilities is neglected. The results of this study call for a review of the (1) current PMDS in light of its goals to improve quality of care and promote patient-centred care, and (2) way it is implemented so as to ensure that the system fully meets its strategic, administrative and development goals without any compromise with its validity and accuracy.

### Recommendations

Based on the findings of this study, the following recommendations are made to improve PM in PHC:

- Review the implementation, validity and accuracy of current PMDS by revisiting discussions on the type of measurements used in PMDS and its systematic implications. Such review should be at a systems level involving all stakeholders and include district level managers as well as all categories of health workers that are subjected to PMDS. It is important that the review should be consultative and promote participation from health managers and providers in all categories. The outcome of this system’s review should outline challenges experienced at all levels that have resulted in the flawed implementation of PMDS. The outcomes of the review should also include a detailed action plan on how to change the current *status quo* and improve the manner in which performance is planned, measured and managed at district level.
- Provide training on PMDS to all personnel who are part of the performance cycle, including nurses nurse managers, performance panel committees, health district management and HR practitioners to improve its implementation. This training should be provided by specialist in the field of PM, change management and coaching for performance. This training should include the following: clarification of the roles and responsibilities of all personnel as suggested by the Public Service Commission – EPMDS 2007 guidelines. It must emphasise strong accountability chains to ensure accountability in line with expressed roles and responsibilities. It should also provide the purpose of a PM system, how performance is measured as well as how to provide and receive feedback and the relevance and benefit of the system.
- The use of PMDS as a tool to identify training needs and to motivate staff should be revisited by health district management. The current emphasis on target-driven results should be broadened to include the quality of care provided by nurses. Through relooking at HRM practices, it is especially important to review the instrument and processes used to measure performance, and whether it is in line with the strategic goals of the healthcare system towards ICSM and PCC. Moreover, there is a need to reconsider the distribution of rewards between monetary and other form of recognition strategies.
- Given the strong culture of compliance rather than active engagement with the PMDS found in this study, there is a need to make changes in the current organisational culture and climate. In this regard, establishment of shared performance goals from district level to facility teams and personal level is recommended. This would require an investigation of readiness to change and interventions that focus on effectively managing change at district level. This could be achieved by providing training to district health managers on transformational leadership and management that is tailored to promote an organisational culture that actively encourages quality healthcare.
- A periodic review (every 3 years) of nurses’ and nurse managers’ perceptions and experiences of PMDS is recommended to provide remedial steps to identify challenges that pose threats to the management of performance at PHC level.

It is further recommended that research be conducted in the following areas: (1) use of PMDS as a tool to promote job satisfaction and enhance other psychological resources such as self-efficacy, emotional intelligence and work engagement; (2) relational leadership as an effective managerial development tool and (3) appraising nurses about practices of person-centred care.

### Limitation of the study

This study used a qualitative research approach, conducted at an NHI pilot site in North West. A purposive sample of professional nurses employed in PHC facilities was selected, which might limit the generalisability of findings to other healthcare facilities in other provinces and district sites that are not NHI pilots.

## Conclusion

The current PMDS needs to be overhauled so as to promote healthy working relationships between nurses and nurse managers to facilitate a collaborative working environment that does not promote individual gains over team capacity. Nurses and nurse managers need to be equipped with the necessary understanding of the value and usefulness of PMDS as well as the skills to implement it properly to ensure that nurses’ contributions are recognised and rewarded appropriately without any favouritism or unfair practices impeding this process. This would allow it to be fully utilised as a valuable managerial tool used to improve health outcomes, identify training and development needs as well as acknowledge hard work and dedication.
